# Fluid-Dependent Single-Frequency Bioelectrical Impedance Fat Mass Estimates Compared to Digital Imaging and Dual X-ray Absorptiometry

**DOI:** 10.3390/nu15214638

**Published:** 2023-11-01

**Authors:** Lexa Nescolarde, Carmine Orlandi, Gian Luca Farina, Niccolo’ Gori, Henry Lukaski

**Affiliations:** 1 Department of Electronic Engineering, Universitat Politècnica de Catalunya, 08034 Barcelona, Spain; 2 Medical Faculty, Tor Vergata University, 00133 Rome, Italy; carmine.orlandi@uniroma2.it; 3 Medical Center Eubion, 00135 Rome, Italy; gianluca.farina@biologo.onb.it; 4 Federazione Italiana Rugby—FIR, Stadio Olimpico, Foro Italico, 00135 Rome, Italy; niccolo.gori@federugby.it; 5 Department of Kinesiology and Public Health Education, University of North Dakota, Grand Forks, ND 58201, USA; henry.lukaski@und.edu

**Keywords:** DXA, BIA, digital imaging, fat mass, extracellular water

## Abstract

The need for a practical method for routine determination of body fat has progressed from body mass index (BMI) to bioelectrical impedance analysis (BIA) and smartphone two-dimensional imaging. We determined agreement in fat mass (FM) estimated with 50 kHz BIA and smartphone single lateral standing digital image (SLSDI) compared to dual X-ray absorptiometry (DXA) in 188 healthy adults (69 females and 119 males). BIA underestimated (*p* < 0.0001) FM, whereas SLSDI FM estimates were not different from DXA values. Based on limited observations that BIA overestimated fat-free mass (FFM) in obese adults, we tested the hypothesis that expansion of the extracellular water (ECW), expressed as ECW to intracellular water (ECW/ICW), results in underestimation of BIA-dependent FM. Using a general criterion of BMI > 25 kg/m^2^, 54 male rugby players, compared to 40 male non-rugby players, had greater (*p* < 0.001) BMI and FFM but less (*p* < 0.001) FM and ECW/ICW. BIA underestimated (*p* < 0.001) FM in the non-rugby men, but SLSDI and DXA FM estimates were not different in both groups. This finding is consistent with the expansion of ECW in individuals with excess body fat due to increased adipose tissue mass and its water content. Unlike SLSDI, 50 kHz BIA predictions of FM are affected by an increased ECW/ICW associated with greater adipose tissue. These findings demonstrate the validity, practicality, and convenience of smartphone SLSDI to estimate FM, seemingly not influenced by variable hydration states, for healthcare providers in clinical and field settings.

## 1. Introduction

Obesity is a multifactorial chronic disease that increases the risk of long-term morbidity, reduces life span, and increases health care costs. More than 650 million people worldwide were identified as obese in 2016, representing a three-fold increase since 1975 [1]. Four million deaths in 2015 were attributed to obesity, and two-thirds were ascribed to cardiovascular disease [2]. The global economic burden of obesity is expected to be 1.2 trillion USD in 2025 [3]. Thus, the availability of reliable and practical methods to identify individuals with excess body fat is a public health need.

Clinicians and health professionals use ranges of body weight relative to height and body mass index (BMI; Wt/Ht2) to stratify adiposity-related health risks in a population. The convenience of BMI in clinical and research settings is offset by its unreliability in estimating body fat and predicting the risk of obesity-related diseases for an individual [3,4,5,6,7,8,9,10]. Because BMI is an insensitive indicator of body composition, it poorly predicts the adiposity of individuals with increased fat-free mass (FFM) [11,12]. Other methods to estimate body composition improve the specificity and precision of estimation of fat and lean body components but are limited by technical complexity, exposure to ionizing radiation, invasiveness, lack of mobility, and cost that prevent their widespread use for routine assessment of body composition [13,14]. Recent reports describe the limitations of BMI and emphasize the need for practical and valid methods to identify overweight and obesity and thus improve the care of patients with excess body fat [15,16,17].

Two methods are amenable to meet this need. Bioelectrical impedance analysis (BIA) relies on the conduction of a safe, administered alternating current to estimate total body water (TBW) [18]. Most BIA methods use 50 kHz phase-sensitive devices and rely on the assumption of constant hydration to estimate FFM and calculate body fat. Alternatively, smartphone two-dimensional standing digital image analysis (2DI) coupled with computational machine learning has recently been used to estimate total body adiposity [19,20,21,22]. Studies reported differences in body fat estimates using smartphone 2DI and BIA compared to DXA but have not provided explanations for the observations [20,21]. Whereas BIA is a valid method to assess TBW [18], and excess body fat increases ECW [23], reliance on the assumption of constant hydration of the FFM may overestimate FFM and, thus, underestimate body fat in adults who are overweight or obese [24,25]. We hypothesize that fluid imbalance is a factor explaining differences in fat estimation with BIA compared to 2DI.

The aim of the present study was to determine the agreement of smartphone single lateral standing digital image (SLSDI) and 50 kHz BIA relative to fat mass (FM) from dual X-ray absorptiometry (DXA) in healthy adults and to determine whether fluid imbalance contributes to any observed differences in FM estimates. 

## 2. Materials and Methods

### 2.1. Participants

We recruited healthy adult Caucasian women and men aged 19 to 64 y using advertisements and word of mouth to participate in this observational study, as all of them are registered patients undertaking nutrition counseling conducted at the Eubion Medical Center and Tor Vergata University in Rome, Italy. Body composition assessments are routinely conducted with counseling. Prospective participants underwent clinical examinations and completed a health questionnaire to establish the absence of an unhealthy condition before participation. Exclusion criteria include metal implants, current treatment for metastatic disease, diabetes, diuretic therapy, or limb loss. This study was strictly observational; therefore, according to the Eubion Center by-law, it does not require review or approval from the ethical committee of the Human Study. Each participant accepted the standard Eubion Center informed consent before participating in any testing.

### 2.2. Body Composition Assessment

Volunteers, wearing form-fitting clothing without jewelry, came to the laboratory after consuming a light meal and emptying their bladders. Standing height and body weight were determined using standard medical equipment (SECA Stadiometer and SECA 762 scale; Hamburg, Germany). 

Body composition estimation included BIA, SLSDI, and DXA administered in random order. Volunteers underwent whole-body BIA testing using a 50 kHz phase-sensitive impedance analyzer that introduced a sinusoidal, constant current (250 µA RMS) (BIVA, EFG 3 Monitor; Akern, Florence, Italy) using a tetrapolar surface electrode placement with paired current-injecting and voltage drop-measuring electrodes (BIAtrodes; Akern, Florence, Italy) separated a minimum of 5 cm and placed on the right wrist and ankle. Volunteers rested supine on a bed without contact with metal for 10 min. This BIA instrument provided direct measurements of resistance (R), reactance (Xc), and phase angle (PhA). The technical accuracy and precision of the BIA instrument were determined following the manufacturer’s recommendations using a supplied calibration circuit referenced as 383 ohm resistance and 45 ohm reactance. We measured the accuracy by comparison of measurements of the calibration circuit with reference values and repeatability as the coefficient of determination and concordance correlation coefficient of duplicate measurements of this circuit to be <2% and 0.98 (*p* < 0.001), respectively. 

Body composition was estimated by using the following BI prediction models.

Kyle et al. [26] (BIA1):

FFM = 4.104 + 0.518 Ht^2^/R + 0.231 Wt + 0.130 Xc + 4.229 Sex * [1 for men and 0 for women]

Sun et al. [27] (BIA2):

Males: FFM = −9.88 + 0.65 Ht^2^/R + 0.26 Wt + 0.02 R

Females: FFM = −11.03 + 0.70 Ht^2^/R + 0.07 Wt + 0.02 R

Units are FFM (kg), height (Ht)^2^/R (cm^2^/Ω), weight (Wt, kg), and Xc (Ω).

**Figure 1:**
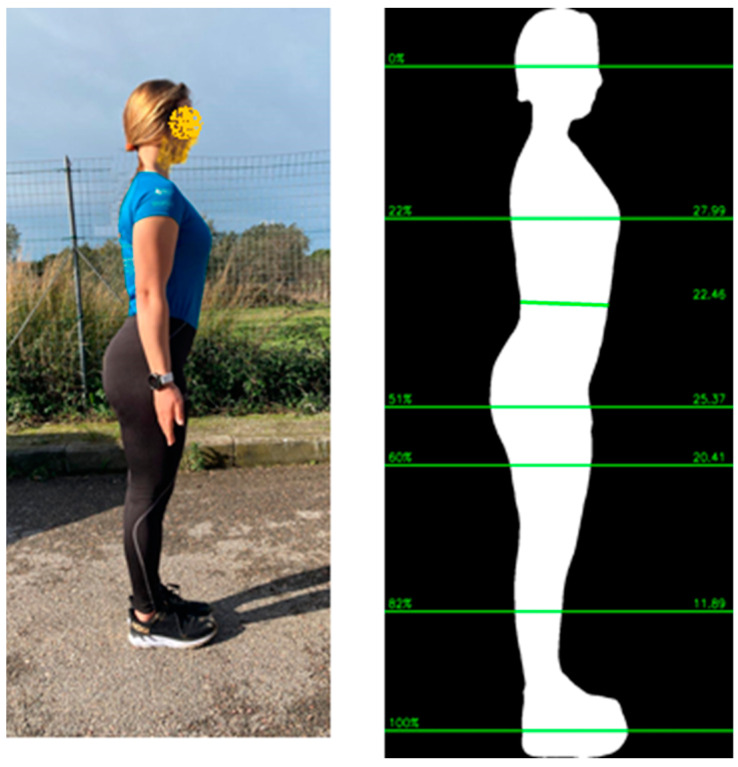
Example of SLDDI photo and silhouette.

A single SLSDI of each volunteer was obtained using the Fit.Your.Outfit (Pixelcando SL-Spain) smartphone APP implemented with a Cloud-based artificial intelligence (AI). An SLSDI of each volunteer was obtained without regard to background or illumination and transformed into an anonymous silhouette [22]. The individual stood upright with the head positioned in the horizontal plane, arms fully extended alongside the body, with feet and legs touching and aligned sagittal to the camera to provide a lateral profile of the body (Figure 1). The smartphone cameras, iOS—iPhone SE or iPhone 11—with CCD resolution greater than 50 megapixels, were either pre-positioned on a stable tripod or held by a second individual who directed the handheld smartphone camera with the lens pointed at the middle of the standing height of each study participant. The Custom Pixelcando version AI 2.3.0 automatically scaled all the digital pictures to a single homogeneous resolution of 5 megapixels and removed any background from the subject silhouette. The distance from the camera to the individual was 1.8 to 2.1 m, which was confirmed by the automatic feedback system of the smartphone APP. The operator downloaded and installed the Fit.Your.Outfit APP, available in iOS and Android from the APP stores, which ensures the high technical quality of digital images for analysis to estimate body composition using proprietary software. The adequacy of the technical quality of the photograph is controlled, and artifacts are prevented by using built-in sensors of modern smart devices and native libraries to detect specific anatomic nodal points to identify parallaxes and improper distance from an individual to a camera and to recognize incorrect arm extension and position, improper alignment of legs and feet, and non-horizontal head position. The quality control protocol provides visual warning instructions to the operator to ensure the acquisition of high-quality digital images for analysis.

Precision, determined as coefficients of determination and concordance coefficients for repeated profiles of SLSDI images and FM estimates, were 0.996 and 0.997 (*p* < 0.0001), respectively, with no difference (<0.1 kg) between repeated images. 

Reference whole-body composition was determined using DXA, a GE Lunar iDXAncore sn 200278, using software version 14.10.022 (Madison, WI, USA). All participants were positioned supine and scanned within the dimensions of the DXA table. Precision estimates were 1.3% for FM and 0.5% for FFM.

Fluid distribution was evaluated using BIA measurements and bioelectrical impedance vector analysis (BIVA) [28]. This method uses tetrapolar BIA and a 50 kHz phase-sensitive impedance device and provides whole-body, direct serial measurements of R, Xc, and phase angle (PhA).

### 2.3. Statistical Methods

Statistical analyses were performed using SYSTAT version 13 (Systat Corporation; San Jose, CA, USA) and version 19.0.3 (MedCalc Software Bv, Ostend, Belgium). Descriptive data are expressed as mean ± SD. Statistical significance was set at *p* < 0.05.

Estimates of FM from the smartphone SLSDI and each BIA prediction model were compared with the reference DXA values. Measurement agreement was evaluated with concordance correlation coefficient (CCC) [29] and standard error of the estimate (SEE) between DXA and each method. Reference and estimates of body fat values in each sex group were compared separately with a paired *t*-test. Bland–Altman plot [30,31] was used to determine bias and limits of agreement (LOA) for the SLSDI and BIA methods compared to DXA determinations.

Effects of fluid distribution were evaluated in a sub-group of males with BMI greater than 25 kg/m^2^, the population indicator for overweight and obesity, to contrast the effects of differences in adipose tissue with an unpaired t-test. Differences between group vector distributions were determined using Hotelling’s T2 test.

## 3. Results

Table 1 describes the physical characteristics of the participants and shows the wide ranges of BMI and FM in the groups. 

The FM values were similar for SLSDI-estimated and DXA-determined FM for females (24.1 ± 11.3 and 24.3 ± 11.6 kg) and males (18.8 ± 7.2 and 18.8 ± 7.6 kg) with no significant difference between methods (Table 2). All 50 kHz BIA equations underestimated (*p* < 0.0001) FM (1.1 ± 2.4 and 3.2 ± 2.4 kg) in females. Two BIA equations overestimated (*p* < 0.0001) FM (1.4 ± 3.9 and 2.9 ± 3.7 kg) in males. The LOA are the range of the differences in FM values determined between DXA, the reference method, and each evaluated method, between which 95% of the differences are expected to fall [30,31]. The LOA for SLSDI were generally smaller than for the BIA prediction equations. 

For females, BIA, compared to SLSDI, had a similar mean absolute error (MAE; 2.2 to 3.4 vs. 2.4 kg) and greater MAE in males (3.3 to 3.6 vs. 2.3 kg). Mean absolute percent error (MAPE) was less with SLSDI than BIA for males (14.8 vs. 20.8 to 21.3%) and similar in females (13.8 vs. 9.9 to 16%).

**Figure 2:**
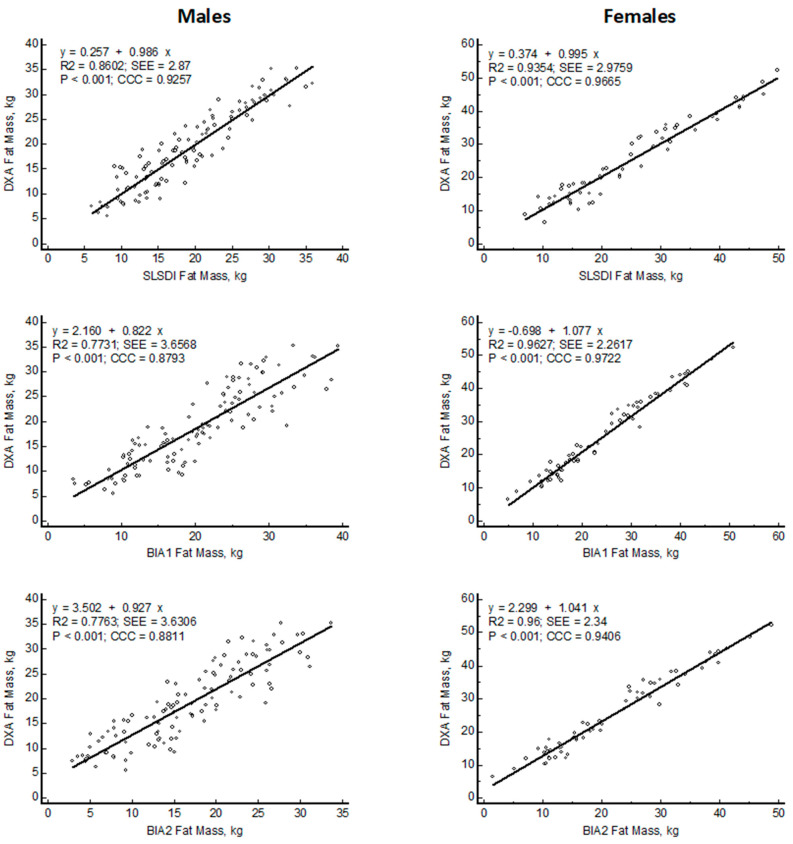
Linear regression plots of fat mass (FM) estimated with smartphone single lateral standing digital image (SLSDI) and 50 kHz bioelectrical impedance equations (BIA) compared to reference dual X-ray absorptiometry (DXA) in males and females.

Figure 2 reveals differences between SLSDI and BIA FM estimates compared to DXA. According to McBride [29], SLSDI shows substantial concordance (0.966) in females and moderate concordance in males (0.925) relative to DXA fat mass. Among females, BIA1 showed substantial concordance (0.972), while BIA2 showed moderate concordance (0.940) with DXA. In contrast, all male samples showed poor CCC (<0.90) between the BIA prediction models and DXA.

The SLSDI predictions of FM for females and males were distributed equivalent with the line of identity with the slopes (0.995 for females and 0.986 for males) the same as 1, and the intercepts (0.3740 for females and 0.2570 for males kg) not different from 0. The SEE values were 2.9759 and 2.8700 kg for females and males, respectively. In contrast, the intercepts for the BIA2 (2.299 kg; *p* < 0.0001) prediction models in females and BIA1 and BIA2 (2.160 and 3.502 kg; *p* < 0.0001) in males were different from 0. The slopes for these lines were variable from 1.041 to 1.077 in females and from 0.822 to 0.927 for males, but not different from 1. The SEE in females (2.3400 kg) was less than the SEE in males (3.6306 kg) (Figure 2).

**Figure 3:**
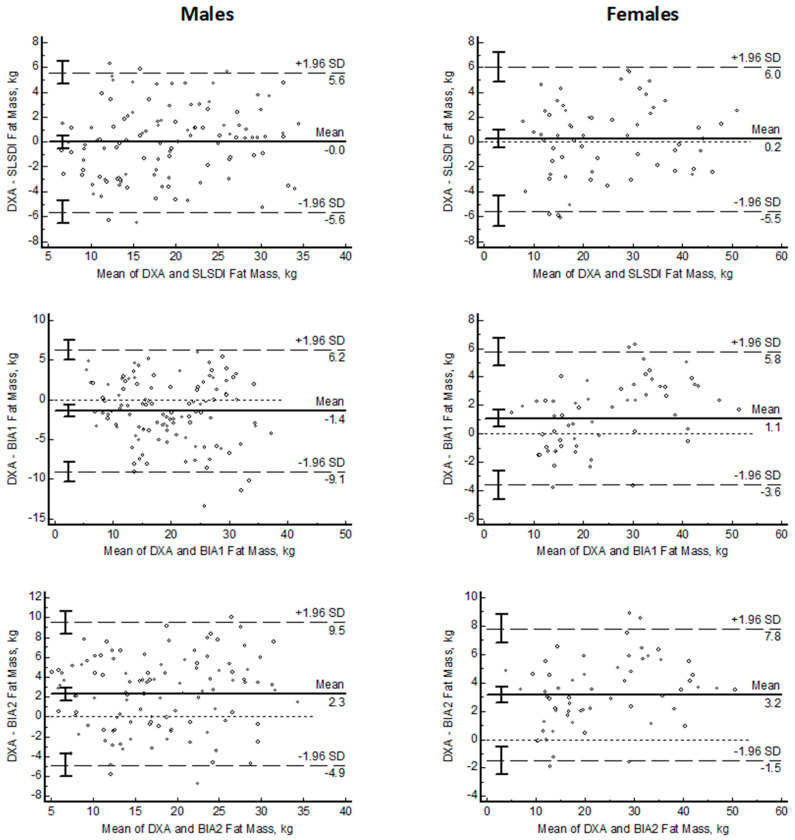
Bland–Altman plots of fat mass estimated with smartphone single lateral standing digital image (SLSDI) and 50 kHz bioelectrical impedance equations (BIA) compared to reference dual X-ray absorptiometry (DXA) in males and females (X = mean DXA and mean method; Y = DXA–method).

Figure 3 shows the Bland–Altman plots of SLSDI and BIA compared to DXA. On average, smartphone SLSDI had similar FM values as DXA FM in females and males (0.2 and 0 kg). Compared to DXA, the BIA predictions of FM had a significant bias (DXA–method) in females (1.1 to 3.2 kg; *p* < 0.0001) and males (1.4 and 2.3 kg; *p* < 0.0001) with one BIA equation overestimating FM (−1.4 kg; *p* < 0.0001). The LOA for SLSDI were smaller compared to the BIA FM estimates. The slopes of the lines relating the differences between DXA and individual methods were not different from 0 for SLSDI in the females and males but were different (*p* < 0.0001) than 0 for one BIA equation in the females and the males.

We tested the hypothesis that excess fat affected fluid balance in men with BMI >25 kg/m^2^ by comparing elite male rugby players with men who were non-rugby players (Table 3). Compared to non-rugby men, male rugby players had greater BMI (30.0 ± 3.3 vs. 28.7 ± 3.0 kg/m^2^; *p* < 0.05) and FFM (84.7 ± 6.6 vs. 68.1 ± 10.1 kg; *p* < 0.0001) with less FM (19.3 ± 6.6 vs. 23.3 ± 6.8 kg; *p* < 0.01) and %fat (18.2 ± 4.7 vs. 25.4 ± 6.2%; *p* < 0.0001).

Estimates of FM using SLSDI were not different from DXA-determined FM in the rugby and non-rugby men (Table 3). Compared to DXA, one BIA equation overestimated FM (−4.0 ± 3.3 kg; *p* < 0.0001) in rugby players, and one BIA model underestimated FM (3.7 ± 3.5; *p* < 0.0001) in non-rugby males. Concordance was moderate between SLSDI and DXA FM and poor for all BIA equations. For both groups, BIA, compared to SLSDI, had a greater MAE (3.0 to 4.3 vs. 2.1 kg and 2.6 to 4.4 vs. 2.6 kg, respectively) and MAPE (15 to 25.8 vs. 13% and 12.5 to 20.6 vs. 12.8%, respectively).

**Figure 4:**
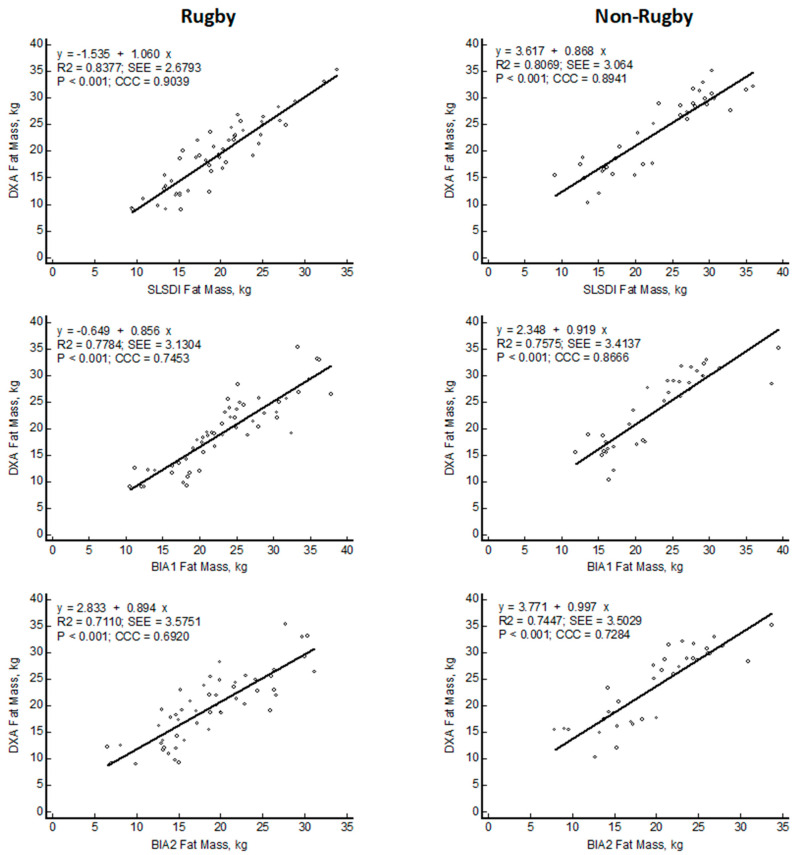
Linear regression plots of fat mass (FM) estimated with smartphone single lateral standing digital image (SLSDI) and bioelectrical impedance equations (BIA) compared to reference dual X-ray absorptiometry (DXA) in rugby and non-rugby men.

Compared to DXA FM values, the SLSDI FM estimates were distributed with slopes not different from 1 and intercepts not different from 0, whereas BIA predictions differed significantly from the line of identity (Figure 4). CCC values also were greater for SLSDI compared to BIA.

**Figure 5:**
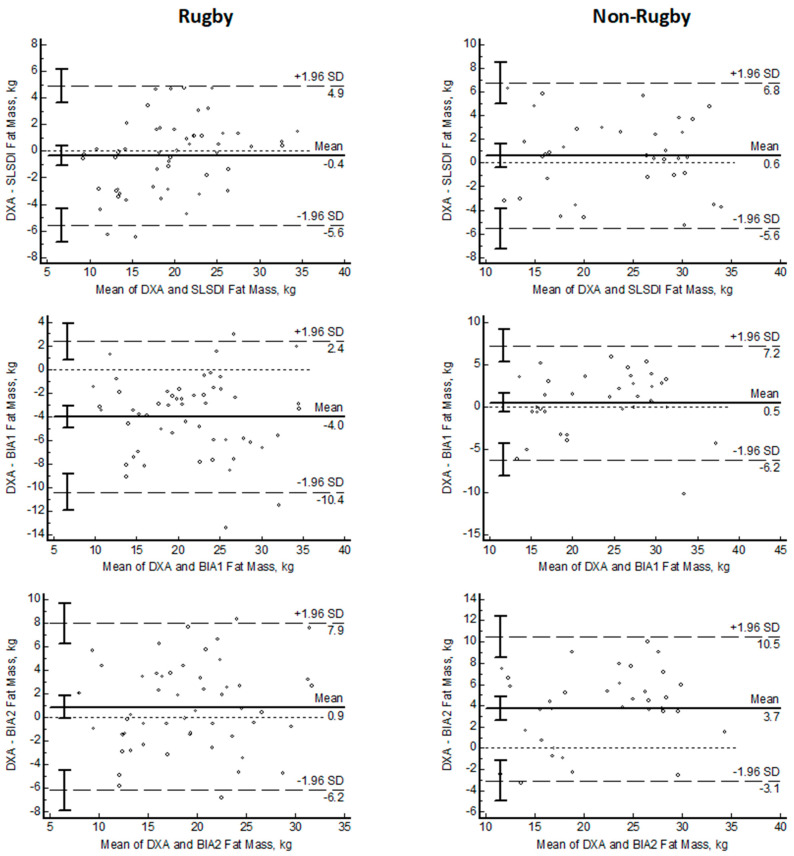
Bland–Altman plots of fat mass estimated with smartphone single lateral standing digital image (SLSDI) and 50 kHz bioelectrical impedance equations (BIA) compared to reference dual X-ray absorptiometry (DXA) in rugby and non-rugby men (X = mean DXA and mean method; Y = DXA–method).

Bland–Altman analysis revealed significant bias between the DXA and the 50 kHz BIA estimates of FM with greater variability of data with BIA equations compared to SLSDI (Figure 5 and Table 3). No bias was detected with SLSDI in either group of men (−0.4; 0.6). Fat mass was significantly overestimated (−4.0 ± 3.3 kg) with one BIA equation in rugby players. One BIA equation significantly underestimated FM (0.9 ± 3.6 kg) in the rugby males and FM in the non-rugby players (3.7 ± 3.5 kg). The slopes of the lines relating the differences between DXA and individual methods were not different from 0 for SLSDI in both groups but were different (*p* < 0.0001) from 0 for the BIA equations in all-male groups. The LOA were greater for all BIA equations compared to SLSDI.

**Figure 6:**
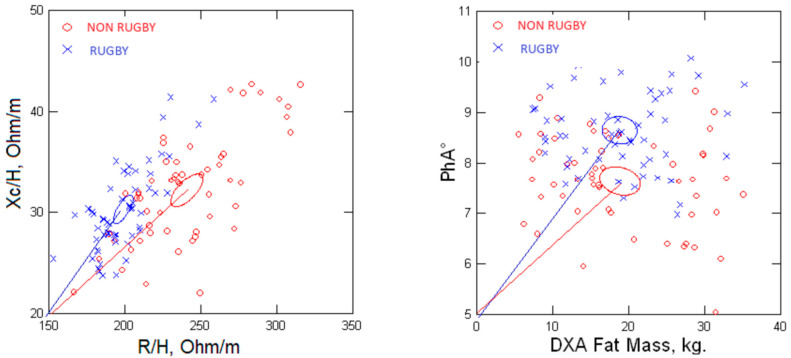
Resistance–reactance (R-Xc) plots showing 95% confidence ellipses (**left** panel) and the effect of fat mass on phase angle (PhA) (**right** panel) for rugby and non-rugby men.

Figure 6 shows the significant group differences (*p* < 0.0001) between the 95% confidence ellipses of men with BMI >25 kg/m^2^. The rugby players had a shorter impedance vector displaced to the left (Z/H = 199.0 ± 7.6 ohm/m), indicating greater conductive mass and structure than the non-rugby males (Z/H = 239.0 ± 31.5 ohm/m). The larger PhA of the rugby players (9.6° ± 0.96°) designates less relative ECW or ECW/ICW, compared to the non-athletic men (8.6° ± 0.82°), associated with the lesser FM. 

## 4. Discussion

Awareness of the limitations of BMI coupled with interest by clinicians and public health researchers for point-of-contact methods to estimate body adiposity is increasing [15,16,17]. Methods with promise need to demonstrate agreement with an accepted reference method. The present study determined the agreement of FM estimated with SLSDI and 50 kHz BIA compared to DXA in healthy adults. Our emphasis was accuracy (differences between DXA and candidate methods or bias) and related measures. The findings demonstrate a high level of accuracy between SLSDI and DXA FM measurements in contrast to a significant underestimation of FM with 50 kHz BIA. On average, bias or the difference between estimated and reference FM values was less with SLSDI (0.1 to 0.2 kg) compared to BIA (1.1 to 3.6 kg). The concordance of FM measurements with DXA was greater for SLSDI than BIA, and the distribution of SLSDI and DXA FM values was similar to the line of identity but significantly different for the BIA FM estimates. Also, the MAE and MAPE of FM estimates, as derived from reference DXA determinations of FM, were less with SLSDI than BIA. Analyses of the differences between DXA and BIA values identified proportional bias (increasing errors with larger FM values) with BIA equations but not SLSDI-estimated FM. 

These findings are generally consistent with other studies comparing 2DI and 50 kHz BIA with reference DXA determinations of body fat [20,21]. Although these studies reported significant differences in body fat between BIA and DXA, they did not identify any factor responsible for the observed differences in FM. We provide the first evidence that fluid imbalance contributes to the errors of BIA in estimating body fat.

A few reports speculated that increased body fat could adversely impact the validity of 50 kHz BIA predictions of FFM [24,25,32,33,34]. The investigators proposed that an expansion of ECW would contribute to an overestimation of FFM but provided no evidence. We evaluated this hypothesis in a subgroup of men with BMI >25 kg/m^2^ with different body composition profiles. Male rugby players, compared to non-rugby playing men, had decreased R/H (specific resistivity [35]) (R/H; 197.0 ± 17.2 vs. 237.2 ± 31.0 ohm/m; *p* < 0.0001) and increased FFM (84.7 vs. 68.1 kg; *p* < 0.001) with decreased FM (19.3 vs. 23.3 kg; *p* < 0.001) and an increase in PhA. The decreased specific resistivity, or increased conductivity, reflects greater TBW and FFM in rugby players. The decreased PhA among the non-rugby men indicates altered fluid distribution in association with the increased body fat and reduced ICW due to less FFM. Increased body fat is associated with an expanded adipose tissue volume that is directly related to an expanded ECW, absolute and relative, previously reported among adults classified as obese using BMI criteria [23,34]. Chemical analyses demonstrated the appreciable average water content of adipose tissue as 15% [36], which contributes to its conductivity but to a lesser degree than muscle. Thus, inter-individual differences in body fat contribute to the variability of ECW in adults.

Raw BIA measurements provide additional support for the finding of increased ECW. The significantly decreased PhA values in the non-rugby men indicate an expansion of ECW based on tracer dilution determinations of fluid volumes and demonstrate an inverse relationship between PhA and ECW/ICW [32]. Also, a decreased PhA has been associated with an expansion of ECW in novice runners with an increased risk of acute kidney injury after a marathon [37]. The findings of imbalanced fluid distribution, ECW/ICW, contribute to the concerns regarding the assumption of constant hydration inherent in the application of BIA to estimate FFM and predict body fat [38,39,40].

The altered fluid balance, characterized by ECW/ICW, significantly affected some 50 kHz BIA predictions of FM. Regardless of fluid status, SLSDI and DXA FM estimates were not different. However, one BIA equation significantly overestimated FM in the rugby group with lower ECW/ICW but significantly underestimated FM in the non-rugby group with increased ECW/ICW. Concordance values among DXA FM values were greater with SLSDI compared to BIA with data distributions of DXA and SLSDI FM similar to the line of identity but significantly different from the BIA FM estimates. 

The independent variables in the 50 kHz BIA prediction models offer potential sources of error in the prediction of FFM. The common predictors of Ht^2^/R and R are significantly related to TBW. Thus, a disproportionate increase in ECW in individuals with excess body fat increases TBW and decreases R, which, assuming constant hydration of FFM, can overestimate FFM and thus underestimate FM. The BIA models also include body weight, which is highly correlated with body fat and can vary depending on environmental, dietary, and physical activity conditions and hence affect hydration. Additionally, the application of 50 kHz BIA prediction equations in groups in whom the original model was not developed can lead to errors. It is well established that body geometry (e.g., limb length, cross-sectional area, and volume) and fluid content (total and distribution) directly affect resistivity and contribute to inter-individual differences in whole body and regional BIA measurements [38,41]. Additionally, regional BIA measurements using different electrode placements, such as foot-to-foot and hand-to-hand, yield discordant estimates of body composition compared to whole-body measurements [42]. These factors contribute to the errors in BIA predictions of body fat using various BIA methods and models and DXA in the present study and other reports [20,21].

Our finding of a high level of agreement between SLSDI and DXA fat measures is consistent with two previous studies that used smartphone 2DI with machine learning and proprietary prediction models to determine body fat in adults [20,21]. Although these studies utilized standing body positions for 2DI, they differed in body positions in relation to the smartphone camera. Nana et al. [20] used front and side positions with 24 replicate digital images for analysis, and Majmudar et al. [21] employed front and back poses. In contrast, the present study utilized a single lateral pose to obtain body contour silhouettes for analysis. The use of multiple body positions and repetitive digital images resulted in greater variability in body fat estimates (±1.5 %fat) compared to 0 in the present study.

This study has some limitations. The interpretation of the expansion of ECW relies on qualitative assessments using 50 kHz BIA. Future studies should incorporate tracer dilution determinations of ECW and TBW and obtain threshold values at which altered fluid distribution affects errors in estimating body fat using BIA. Additionally, investigators should determine the effect of graded body fat levels on errors relative to criteria reference methods, particularly in adults with sarcopenic obesity. Evaluation of the model of Chamney et al. [43] using bioelectrical impedance spectroscopy, which proposes to discriminate normally hydrated FFM and AT from excess ECW, may reduce the errors in estimating FM associated with expanded ECW in individuals with variable levels of FM. Findings in non-dialysis patients with chronic kidney disease demonstrated poor agreement between this model and DXA, with an overestimation of FFM and an underestimation of FM [44]. Thus, further research to determine the accuracy of these different BIA methods is needed.

## 5. Conclusions

Smartphone SLSDI provides highly comparable DXA estimates of FM of adults with a wide range of body fat. The agreement of SLSDI in the estimation of FM compared to DXA, coupled with the convenience, practicality, and cost-efficiency, facilitate an innovative method to assess body composition to enable routine assessment of body fat for healthcare personnel in many environments and for biomedical researchers outside the laboratory. Importantly, the SLSDI method does not require specialized equipment, only generally available smartphones. It surmounts the limitations of BMI as an index of body fat [15,16,17] and provides a novel method to estimate abdominal FM [22]. Moreover, SLSDI image-based FM estimates provide overall better performances than impedance-based methods because they are seemingly not influenced by variable hydration states.

## Figures and Tables

**Table 1:** Physical characteristics of the study participants. Values are mean ± SD (min-max).

	Females	Males	Rugby and Non-Rugby Males	
			BMI > 25 kg/m²	
	Total	Total	Rugby	Non-Rugby
**N**	69	119	54	40
**Age, y**	37 ± 13	28 ± 9	26 ± 3	36 ± 15
	(19–65)	(19–68)	(20–34)	(19–68)
**Height, cm**	162.7 ± 6.3	182.5 ± 8.7	186.2 ± 7.2	179.7 ± 9.2
	(150.0–178.0)	(160.0–202.0)	(173.0–202.0)	(163.0–198.0)
**Weight, kg**	67.8 ± 14.5	93.2 ± 13.3	104.8 ± 11.4	91.4 ± 12.2
	(41.8–103.6)	(61.1–125.6)	(79.4–123.0)	(69.5–124.6)
**BMI, kg/m^2^**	25.6 ± 5.4	27.6 ± 4.0	30.4 ± 3.3	28.7 ± 3.0
	(16.1–37.2)	(19.5–37.0)	(25.2–36.6)	(25.1–37.1)
**DXA Fat, kg**	24.3 ± 11.6	18.8 ± 7.7	19.3 ± 6.5	23.3 ± 6.9
	(6.4–52.3)	(5.6–35.2)	(9.0–35.2)	(10.3–35.1)
**DXA Fat, %**	34.2 ± 10.2	19.8 ± 6.7	18.2 ± 4.7	25.4 ± 6.2
	(12.1–50.5)	(8.3–36.8)	(9.8–28.9)	(11.4–36.8)
**DXA FFM, kg**	43.5 ± 5.6	74.4 ± 12.7	84.7 ± 6.6	68.1 ± 10.1
	(31.0–55.8)	(47.4–103.2)	(68.9–103.2)	(50.6–97.2)

N: sample size; BMI: body mass index; DXA: dual X-ray absorptiometry; and FFM: fat-free mass.

**Table 2:** Levels of agreement for body fat estimates using smartphone single lateral standing digital image and 50 kHz bioelectrical impedance analysis compared to dual X-ray absorptiometry. Values are mean ± SD.

		Males N = 119	Females N = 69
**DXA**	Fat mass, kg	18.8 ± 7.6	24.3 ± 11.6
**SLSDI**	Fat mass, kg	18.8 ± 7.2	24.1 ± 11.3
	Bias, kg	−0.1 ± 2.9	0.2 ± 3.0
	p	0.97	0.29
	SEE, kg	2.9	3.0
	CCC	0.93	0.96
	MAE, kg	2.3 ± 1.7	2.4 ± 1.6
	MAPE, %	14.8 ± 13.3	13.8 ± 14.3
	LOA 95% CI, kg	5.6–(−5.6)	6.0–(−5.5)
**BIA1**	Fat mass, kg	20.3 ± 8.2	23.2 ± 10.6
	Bias, kg	−1.4 ± 3.9	1.1 ± 2.4
	p	0.0001	0.0001
	SEE, kg	3.7	2.3
	CCC	0.86	0.98
	MAE, kg	3.3 ± 2.5	2.2 ± 1.4
	MAPE, %	20.8 ± 18.3	9.9 ± 6.2
	LOA 95% CI, kg	6.2–(−9.1)	5.8–(−3.6)
**BIA2**	Fat mass, kg	21.1 ± 7.3	21.1 ± 10.9
	Bias, kg	2.3 ± 3.7	3.2 ± 2.4
	p	0.0001	0.0001
	SEE, kg	3.6	2.3
	CCC	0.84	0.94
	MAE, kg	3.6 ± 2.4	3.4 ± 2.1
	MAPE, %	21.3 ± 15.8	16.0 ± 13.9
	LOA 95% CI, kg	9.5–(−4.9)	7.8–(−1.5)

DXA = dual X-ray absorptiometry; SLSDI = single lateral standing digital image [22]; BIA1 = Kyle et al. [26]; BIA2 = Sun et al. [27]; Bias = DXA–method; p = probability value; SEE = standard error of estimate; CCC = concordance correlation coefficient; MAE = mean absolute error; MAPE = mean absolute percent error; LOA = limits of agreement (DXA–method); and CI = confidence interval.

**Table 3:** Levels of agreement for body fat estimates using smartphone single lateral standing digital image and 50 kHz bioelectrical impedance analysis compared to dual X-ray absorptiometry of men with body mass index greater than 25 kg/m². Values are mean ± SD.

		Rugby N = 54	Non-Rugby N = 40
**DXA**	Fat mass, kg	19.3 ± 6.6	23.3 ± 6.8
**SLSDI**	Fat mass, kg	19.7 ± 5.7	22.7 ± 7.1
	Bias, kg	−0.4 ± 2.7	0.6 ± 3.2
	p	0.33	0.22
	SEE, kg	2.6	3.1
	CCC	0.92	0.89
	MAE, kg	2.1 ± 1.7	2.6 ± 1.8
	MAPE, %	13.0 ± 14.2	12.8 ± 10.5
	LOA 95% CI, kg	4.9–(−5.6)	6.8–(−5.6)
**BIA1**	Fat mass, kg	23.4 ± 6.8	22.8 ± 6.5
	Bias, kg	−4.0 ± 3.3	0.5 ± 3.4
	p	0.0001	0.36
	SEE, kg	3.1	3.4
	CCC	0.78	0.87
	MAE, kg	4.3 ± 2.9	2.6 ± 2.2
	MAPE, %	25.8 ± 21.2	12.5 ± 12.4
	LOA 95% CI, kg	2.4–(−10.4)	7.2–(−6.2)
**BIA2**	Fat mass, kg	18.5 ± 6.2	19.7 ± 5.9
	Bias, kg	0.9 ± 3.6	3.7 ± 3.5
	p	0.08	0.0001
	SEE, kg	3.5	3.5
	CCC	0.85	0.73
	MAE, kg	3.0 ± 2.1	4.4 ± 2.5
	MAPE, %	16.8 ± 13.1	19.1 ± 11.0
	LOA 95% CI, kg	7.9–(−6.2)	10.5–(−3.1)

DXA = dual X-ray absorptiometry; SLSDI = single lateral standing digital image [22]; BIA1 = Kyle et al. [26]; BIA2 = Sun et al. [27]; Bias = DXA method; p=probability value in comparison with DXA; SEE = standard error of estimate; CCC = concordance correlation coefficient; MAE = mean absolute error; MAPE = mean absolute percent error; LOA = limits of agreement; and CI = confidence interval.

## Data Availability

The data supporting this study’s findings are available from the corresponding author upon reasonable request.
